# XEN Glaucoma Implant for the Management of Glaucoma in Naïve Patients versus Patients with Previous Glaucoma Surgery

**DOI:** 10.3390/jcm10194417

**Published:** 2021-09-26

**Authors:** Katarzyna Lewczuk, Joanna Konopińska, Joanna Jabłońska, Jacek Rudowicz, Patrycja Laszewicz, Diana Anna Dmuchowska, Zofia Mariak, Marek Rękas

**Affiliations:** 1 Department of Ophthalmology, Military Institute of Medicine, 04-141 Warsaw, Poland; klewczuk@gmail.com (K.L.); joannajablonska.md@gmail.com (J.J.); jrudowicz@wim.mil.pl (J.R.); plaszewicz@wim.mil.pl (P.L.); rekaspl@gmail.com (M.R.); 2 Department of Ophthalmology, Medical University in Bialystok, M. Sklodowska-Curie 24A STR, 15-276 Bialystok, Poland; diana_anka@op.pl (D.A.D.); mariakzo@umb.edu.pl (Z.M.)

**Keywords:** refractory glaucoma, XEN implant, management, retrospective study, minimally invasive glaucoma surgery, glaucoma surgery, bleb, subconjunctival stent

## Abstract

This retrospective study analyzed the surgical and refractive outcomes of a XEN Gel Implant (Allergan, Abbvie Company, Irvine, CA, USA) in naïve patients versus those with previous glaucoma surgery. We evaluated the efficacy of XEN implantation in 86 glaucoma patients during a long-term follow-up period. Patients were divided into two groups: naïve patients (Group 1) and patients with previous glaucoma surgery (Group 2). Eyes that received a XEN Gel Stent placement from December 2014 to October 2019 were included. Intraocular pressure (IOP) change, corrected distance visual acuity (CDVA), change in glaucoma medications, frequency of slit lamp revision procedures, and frequency of secondary glaucoma surgeries were the primary outcomes. In Group 1, the mean IOP before surgery was decreased significantly from 25.00 ± 7.52 mmHg to 16.83 ± 5.12 mmHg by the end of the study. In Group 2, the mean IOP decreased significantly from 25.35 ± 7.81 mmHg to 17.54 ± 5.34 mmHg. The mean IOP decrease from baseline was 29% in Group 1 and 27% in Group 2 (*p* = 0.567). There were no significant differences between the groups in the IOP baseline level, the final level, or the change between preoperative and final levels. The qualified success rate for Group 2 was 68.7% versus 76.5% for Group 1 for the initial procedure and 15.4% vs. 20.2%, respectively, for complete success rate (*p* > 0.05). However, at the end of the follow-up, more patients achieved an IOP < 18 mmHg in Group 1 than in Group 2. Despite the need for more anti-glaucoma medications, repeat XEN Gel implantation appears to show promising results in patients with previously failed anti-glaucoma procedures, owing to its minimal invasiveness.

## 1. Introduction 

Glaucoma remains the second leading cause of blindness worldwide [1]. By 2040, there will be an estimated 111.8 million people with glaucoma globally [1]. At present, the only known intervention that can slow the progression of this disease is the reduction of intraocular pressure (IOP) to prevent loss of retinal nerve fibers and subsequent damage to the visual field. Refractory glaucoma poses a challenge for glaucoma surgeons [2,3]. It is defined as uncontrolled IOP with associated visual field deterioration despite maximum tolerated anti-glaucoma treatment and previously unsuccessful anti-glaucoma procedures [4]. The most common causes of surgical failure are subconjunctival and episcleral fibrosis. The progressive fibrosis of subconjunctival and episcleral tissues has been attributed to the stimulation of an increased number of subepithelial conjunctival fibroblasts after conjunctival surgery. Although previous filtration surgery is a known risk factor for trabeculectomy failure, repeat trabeculectomy is frequently performed in cases of initial trabeculectomy failure [5]. Trabeculectomy is a standard filtration procedure that results in the lowering of IOP to 12.7 ± 5.8 mm/Hg at 1 year after an initial procedure and to 13.5 ± 5.3 mm/Hg after a repeated procedure [6]. Unfortunately, it also has the highest rate (78%) of overall complications. Moreover, a repeat trabeculectomy is less effective than the initial trabeculectomy [7]. In eyes that underwent a repeat trabeculectomy, younger age and the requirement for laser suture lysis were significant risk factors for surgical failure. The need for medication was higher after repeated trabeculectomies, as opposed to the initial trabeculectomies. It is also known that eyes that undergo an initial trabeculectomy require a statistically lower number of medications than eyes that undergo a repeat trabeculectomy [7].

In 2016, the US Food and Drug Administration approved the new XEN Gel Stent implant (XEN 45 Gel Stent) for the treatment of open-angle glaucoma and refractory glaucoma (CE certified in 2011). This implant belongs to the minimally invasive glaucoma surgery category. It is a 6-mm-long hydrophilic tube with an internal diameter of 45 µm, and its mode of action, similar to that of trabeculectomy and glaucoma draining devices (GDD), creates a new route of outflow for the aqueous humor to exit the anterior chamber, bypassing the potential site of increased outflow resistance in the Schlemm canal while using the subconjunctival outflow route. Moreover, similar to trabeculectomy, it is a filtration-bleb-dependent procedure [8]. Its usage is comparable with that of trabeculectomy, which is considered the gold standard in glaucoma surgery [9,10]. However, in contrast to trabeculectomy, the advantages of XEN Gel Stent implants include microinvasive ab interno/ab externo access, sparing of the sclera and conjunctiva, obviating the need for iridectomy and sutures, and a shorter procedure time. Studies that compared the two treatments demonstrated similar hypotensive efficacy with a better safety profile for XEN [11,12,13,14,15,16,17,18].

The aim of this study was to compare the efficacy and safety of XEN Gel Stent implantation in naïve patients versus those who had previously undergone glaucoma surgery. Therefore, this study retrospectively evaluated the efficacy of XEN implantation in 86 glaucoma patients during a long-term follow-up period.

## 2. Materials and Methods

This study was performed with the approval of the Bioethics Committee of the Military Institute in Warsaw, in accordance with the ethical standards as laid down in the 1964 Declaration of Helsinki and its later amendments, or comparable ethical standards. Study subjects provided written informed consent for ophthalmological examination and the use of their clinical data for publication on the day of the first ophthalmological examination. This study followed the STROBE guidelines [19].

We retrospectively analyzed the data of consecutive patients who underwent XEN implantation for different types of glaucoma, defined as IOP ≥ 21 mmHg. We enrolled patients fulfilling the criteria for the two groups. In Group 1, we included eyes with no previous anti-glaucoma surgeries, while in Group 2, we included patients with a history of one to six previous unsuccessful anti-glaucoma procedures. Surgeries were performed by the same experienced surgeon (MR) from December 2014 to October 2019. The inclusion criteria were as follows: (1) age ≥ 18 years, (2) progression of glaucoma in visual field examination, and (3) failure to achieve target IOP with maximally tolerated topical IOP-lowering treatment or intolerance to drugs. Primary and secondary open-angle glaucoma cases were included in this study. Only solo procedures (without concomitant or previous cataract removal) were included in the study. Other inclusion criteria were the presence of healthy mobile conjunctiva in at least one quadrant and corrected distance visual acuity (CDVA) better than light perception. The exclusion criteria were as follows: shallow anterior chamber and angle-closure glaucoma, presence of clinically significant inflammation or infection within 30 days before surgery, history of corneal refractive surgery, corneal deposits or haze preventing intraoperative viewing of the anterior chamber, advanced age-related macular degeneration, known or suspected allergy or sensitivity to porcine products or glutaraldehyde, pregnant or nursing women, and lack of consent to participate in the study. If patients were taking anticoagulants before surgery, they were discontinued under the supervision of a general practitioner, changed to low-molecular-weight heparin injections perioperatively, and then continued after surgery. If both eyes were eligible for surgery, the eye with the worse CDVA and/or visual field was operated on first. The number of previous surgical procedures was not an exclusion criterion.

During the procedure, depending on the availability of the surgical access, the surgeon used the ab interno or ab externo technique, following previously described guidelines [20,21]. According to recent research, both approaches have similar efficacies on IOP [22]. In cases where only the lower quadrants were accessible, ab externo access was the technique of choice. Treatments were performed with 40 µg of mitomycin C, which was injected under the conjunctiva at least 6 mm from the corneal limbus in the projection of the future filtering bleb (Video S1). The eyes were treated postoperatively with topical medication containing steroids (loteprednol; TID for 4 weeks, which was then tapered to BID for 1 week), an antibiotic (moxifloxacin; TID for 2 weeks), and nonsteroidal anti-inflammatory drugs (diclofenac; TID for 4 weeks) [21].

At the preoperative visit, the following information was obtained from the patient: age, sex, previous surgical procedures, CDVA (according to the Snellen chart), IOP (measured using Goldmann applanation tonometry), mean deviation of the visual field (assessed using the 24-2 algorithm of the Humphrey visual field test), and the number of IOP-lowering medications used (counted as single topical substances or oral acetazolamide). 

The primary outcome measure was complete surgical success defined as a 20% decrease in IOP or IOP of ≤18 mmHg without medication. Qualified success was defined as a 20% decrease in IOP or IOP ≤ 18 mmHg with up to two anti-glaucoma medications.

The number of anti-glaucoma medications, IOP, and CDVA was also analyzed before surgery and at 1 day, 1 week, 1 month, 3 months, 6 months, 1 year, and 2 years postoperatively. From the day of the surgery, the patients discontinued all anti-glaucoma drugs, which were restarted if the target IOP was not achieved after surgery, according to the Advanced Glaucoma Intervention Study rule [23]. The final mean difference (MD) values at the end of the follow-up period were compared with those evaluated at the first visit. 

Moreover, 5-fluorouracil (5-FU) injections, transconjunctival needling, and subsequent anti-glaucoma surgeries were recorded as they occurred. Additional procedures were applied when the following criteria were met: 5-FU injection (5 mg in 0.2 mL), progressive increase in the IOP greater than 16 mmHg, and the development of subconjunctival fibrosis (manifested as engorged and tortuous blood vessels above the scleral flap); for needling, diagnosis of fibrosis (based on the abovementioned clinical signs), insufficient subconjunctival outflow, increase in IOP, or flattening of the bleb. Injections of 5-FU were administered for 5 consecutive days or until the fibrosis was abated and the IOP stabilized, provided that no anti-metabolite-related adverse effects occurred [24]. The number of complications, such as hypotony, choroidal detachment, corneal edema and keratopathy, improper positioning, leakage of the filtering bleb, implant displacement or occlusion, bleeding into the anterior chamber, malignant glaucoma, and intraocular inflammation were noted. Hypotony was defined as IOP ≤ 5 mmHg in two consecutive measurements at any stage of the follow-up period. 

The sample size was determined assuming the baseline IOP was at the level of 25 mmHg. In order to detect a decline of 5 ± 5 mmHg, i.e., a 20% decline at the end of the study, with 90% power and 5% significance level, at least 17 patients were needed (assuming a 20% loss to follow-up) [25]. If any follow-up evaluation was missed (in this study, two patients died and four were lost to follow-up), the performance outcomes were based on multiple imputations for the missing values [26]. For 3 eyes from Group I and 5 from Group II, further surgical treatment due to unsatisfactory reduction of the IOP was required. A trabeculectomy was performed in 2 eyes and transscleral cyclophotocoagulation was performed for 6 eyes. The follow-up of these patients ended at the moment of their qualification for another IOP-reducing surgical procedure.

Statistical analysis was performed using R software, version 4.0.5 (R Foundation for Statistical Computing, Vienna, Austria). The study variables are presented using descriptive statistics. The normality of the distribution of quantitative variables was assessed using the Shapiro-Wilk test, data skewness, and kurtosis indicators, as well as visual assessment of the histograms. The equality of variance was checked using Bartlett’s test. Group comparisons were performed with Fisher’s exact test or chi-square test for nominal variables and the independent *t*-test or Mann-Whitney *U* test for continuous variables. A comparative analysis of the results between the beginning and end of the study was performed using the paired *t*-test. The MD with a 95% confidence level (CI) was also calculated. Additionally, the cumulative incidence of complete success and cumulative incidence of qualified success were calculated using the Kaplan-Meier survival analysis. The log-rank chi-square test was used to compare the complete and qualified success rates between the groups. Missing values were omitted when analyzing individual variables. A significance level of α = 0.05 was used, and all tests were two-sided.

## 3. Results

### 3.1. Demographics and Glaucoma History

Eighty-six eyes of 86 subjects were included in the study with 43 eyes in the group of naïve patients (further described as Group 1) and 43 eyes in the group with previous surgeries (Group 2). There were no significant differences between the groups in terms of sex and age distribution. The duration of glaucoma differed significantly between groups, with a longer timespan in Group 2 (18.18 ± 14.88 years) than in Group 1 (10.03 ± 10.86), *p* = 0.008. The median number of prior procedures in Group 2 was 2 (range, 1–6), *p* < 0.001 (Table 1). Prior procedures included trabeculectomy (69%), sclerectomy (55%), cyclodestructive procedures (41%), canaloplasty (23%), ExPress seton implantation (11%), Ahmed glaucoma valve implantation (8%), and a history of laser trabeculoplasty (69%). In addition, the following procedures with potential conjunctival scarring were performed: 23-G vitrectomy with silicone oil in 19% and scleral buckling in 15% of patients.

The CDVA before surgery was 0.37 ± 0.28 for Group 1, which improved to 0.44 ± 0.36 (MD, 0.08; 95% CI: 0.02, 0.13; *p* = 0.011) by the end of the follow-up period. For Group 2, the CDVA before surgery was 0.38 ± 0.31, which increased to 0.45 ± 0.36 (MD, 0.07; 95% CI: 0.01, 0.14; *p* = 0.024) by the end of the follow-up period. There were no significant differences between the two groups in the CDVA baseline level, the final level, or change between preoperative (pre-op) and final levels.

In Group 1, the mean IOP before surgery was 25.00 ± 7.52 mmHg, which decreased significantly to 16.83 ± 5.12 mmHg by the end of the study (MD, −6.59; 95% CI: −10.96, −5.73; *p* < 0.001), while for Group 2, the IOP decreased significantly from 25.35 ± 7.81 mmHg to 17.54 ± 5.34 mmHg (MD, −8.00; 95% CI: −10.87, −5.13; *p* < 0.001). The mean decrease from baseline was 29% in Group 1 and 27% in Group 2. There were no significant differences between the groups in the IOP baseline level, the final level, or change between preoperative and final levels (Table 2).

**Figure 1:**
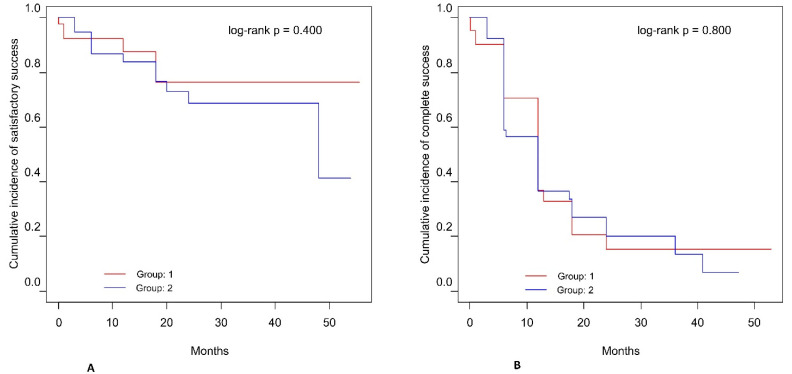
Kaplan-Meier cumulative incidence of qualified success and complete success. Qualified success was defined as a decrease of 20% in IOP, or IOP ≤ 18 mmHg with or without medication (**A**). Complete surgical success was defined as a decrease of 20% in IOP, or IOP ≤ 18 mmHg without medication (**B**).

The Kaplan-Meier cumulative incidence rate of qualified success after 24 months was 76.5% (95% CI: 60.7%, 96.5%) for Group 1 and 68.7% (95% CI: 53.9%, 87.6%) for Group 2 (Figure 1). The cumulative incidence rate of complete success after 2 years of observation was 15.4% (95% CI: 6.1%, 39.1%) for Group 1 and 20.2% (95% CI: 10.2%, 39.9%) for Group 2. There was no significant difference in qualified/complete success between Groups 1 and 2, based on the chi-square log-rank test (*p* = 0.400 for qualified success and *p* = 0.800 for complete success).

At the end of the follow-up period, a decrease in CDVA, compared to the baseline value, was observed in 17% of eyes in Group 1 and in 21% of eyes in Group 2, while an increase was observed in 60% and 59% of eyes in Group 1 and Group 2, respectively, both of which were not significant (*p* = 0.878). However, a decrease in IOP level was observed in 88% of eyes in Group 1 and 80% of eyes in Group 2, and an increase in IOP was noted in 7.3% and 18% of patients from both groups, respectively, but was not significant (*p* = 0.383). A decrease in IOP greater than or equal to 20% occurred in 66% of patients in Group 1 and 56% of patients in Group 2 (*p* = 0.492). A final IOP level of less than 18 mmHg affected 85% of eyes in Group 1 vs. 62% of eyes in Group 2 (*p* = 0.030).

### 3.2. Additional Interventions and Procedures

A massage was recommended to 35% of patients in Group 1 and 21% of patients in Group 2, and needling was performed in 70% and 74% of patients in both groups, respectively, although the differences were not statistically significant. There were no differences between the groups in the number of needlings, preoperative and final number of drugs, and time to administration of the first drug. Additional interventions and procedures were performed in 35% of the patients in Group 1 vs. 23% of patients in Group 2 (*p* = 0.342). Complications occurred in 23% of patients in Group 1 and 30% in Group 2 (*p* = 0.626), and reoperations were performed in 17% and 32% of patients in both groups, respectively (*p* = 0.309) (Table 3).

### 3.3. Postoperative Complications

The intaoperative and postoperative complications have been presented in the Table 4.

None of the phakic patients developed cataract during the follow-up period.

### 3.4. Visual Field

Preoperatively, MD on visual field testing was −14.51 ± 7.3 dB in Group 1 and −16.9 ± 15.5 dB in Group 2 (*p* = 0.12). After 24 months of observation, MD in Groups 1 and 2 were −15.2 ± 10.4 dB and −17.8 ± 8.9 dB (*p* = 0.35), respectively. Stabilization of the visual field was observed in 83.3% of patients in Group 1 and in 79.7% of patients in Group 2 (*p* = 0.14). Deterioration of mean deviation was found in 16.7% of patients in Group 1 and in 20.3% of patients in Group 2 (*p* = 0.24).

## 4. Discussion

XEN Gel Stent implantation is becoming increasingly popular among ophthalmic surgeons. However, to the best of our knowledge, no clinical studies have compared the safety and efficacy of this technique regarding the number of previous anti-glaucoma surgeries. This retrospective analysis demonstrates that implantation of XEN as the first antiglaucoma procedure has a similar efficacy and safety profile to that of implantation as the next procedure. Reduction in IOP and glaucoma medications were comparable in magnitude between the groups, with a tendency toward superiority in Group 1.

The failure of filtration surgery is most likely caused by the breakdown of the blood-aqueous barrier and subsequent greater wound-healing response. The conjunctiva altered by previous surgery has increased subepithelial cellularity, which causes a subsequent greater wound-healing response. This leads to the production, contraction, and remodeling of collagen tissue, causing scarring at the conjunctival-episcleral interface. The use of antimetabolites reduces the risk of failure, but in 15–38.8% of cases, the target pressure level is not achieved [27,28].

In a retrospective case series of repeated trabeculectomy (113 eyes), Jagannathan et al. [5] demonstrated a success rate of 53.1% in cases with an IOP of <17 mmHg after 1 year of follow-up. In another study, Law et al. [7] compared the outcomes of repeat trabeculectomy (50 eyes) versus primary trabeculectomy (50 eyes) in primary open-angle glaucoma at 3 years follow-up. In their study, a success rate of 32% versus 52% (*p* = 0.021) was observed for an IOP of 12 mmHg. For the less stringent IOP reduction criteria (IOP < 18 mmHg), the differences were not statistically significant (54.6% vs. 68%, *p* = 0.131). In a prospective trial, Canayaka et al. [17] found minimal differences between the success rate in repeat (28 eyes) and primary trabeculectomy (59 eyes). The success rate was 55.8% versus 60.8% (*p* = 0.46) at 2 years follow-up. In this context, we demonstrated the qualified success rate for repeat XEN Gel procedure of 68.7% versus 76.5% for the initial procedure (for complete success, 15.4% vs. 20.2%, respectively), with no significant difference in qualified/complete success between Groups 1 and 2. However, at the end of follow-up, more patients achieved IOP < 18 mmHg in the group with the initial procedure than in the group with the repeat procedure (85.4% vs. 61.5%, *p* = 0.03).

Karimi et al. [27] assessed 17 cases of XEN Gel implantation as a secondary glaucoma procedure after a failed trabeculectomy; we obtained similar results. Preoperatively, the IOP decreased from 21.5 ± 2.4 mmHg to 13.6 ± 3.4 mmHg at the end of follow-up (12 months). The most frequent adverse events were hypotony and IOP spikes, which occurred in 23.5% and 11.8% of cases, respectively. A subsequent filtration procedure was required in 11.8% of cases. 

Hengerer et al. [29] compared XEN 45 solo (*n* = 200) with XEN 45 combo (*n* = 39) in a retrospective single-center single-surgeon analysis of 117 patients with open angle glaucoma (48.3%), 62 with pseudoexfoliative glaucoma (25.6%), 21 with primary angle-closure glaucoma (8.7%), 14 with NVG (5.8%), and 10 with inflammatory glaucoma (4.2%) with a 12-month follow-up period. A total of 170 eyes (70.2%) were subjected to prior intervention, with 53 eyes (21.9%) having a prior iStent implant and 52 eyes (21.5%) having undergone prior trabeculectomy. The mean initial intraocular pressure in the XEN solo group was 31.5 ± 8.4 mmHg, with 3.1 ± 1.0 medications, decreasing to 14.3 ± 4.2 mmHg with 0.3 ± 0.7 medications; that in the XEN combo group was 35.7 ± 12.0, with 3.3 ± 1.0 medications, decreasing to 13.9 ± 2.5 mmHg for 0.4 ± 0.7 medications at postoperative 12-month follow-up. Complete success was achieved in 55.4% of the eyes and qualified success in 73%, with no significant difference between the two groups. This is in line with our results; however, to uniform our group, we analyzed only solo procedures.

The clinical trial of Grover et al. [30] demonstrated that 75.4% of patients with RG achieved a 20% IOP-lowering effect from baseline on the same number or fewer medications at 12 months follow-up. This satisfactory result may be attributed to a more homogenous group, since the one of exclusion criteria was previous vitreoretinal surgeries.

There are several limitations to this study. First, this is a small, retrospective research, thereby affecting the generalizability of the study findings. Second, the assessment of patients, including IOP monitoring, was not masked and therefore subject to observer bias. Third, our control group was characterized by extremely diverse cases and different mechanisms of glaucoma in different patients. Lastly, our study focused only on Caucasian populations. While this may be a limitation of the study, the patient population was homogeneous and related to a specific ethnic group. However, to our knowledge, no previous studies comparing the initial and repeat XEN Gel implantation with comparable study groups are available.

## 5. Conclusions

Our study showed that the surgical success rate after XEN^®^ Gel Stent implantation as an initial procedure did not differ from that in patients with previous anti-glaucoma surgeries. In summary, repeat XEN Gel implantation might also be beneficial for patients with severe cases who had previously undergone multiple glaucoma surgeries. Pharmacoeconomic and quality-of-life studies in different groups of glaucoma patients with longer follow-up are necessary to confirm the efficacy of this treatment method as an alternative to trabeculectomy.

## Figures and Tables

**Table 1:** Baseline characteristics of the study group.

	Group 1 No Previous Surgery (*n* = 43)		Group 2 1 or More Previous Surgeries (*n* = 43)		*p*
	*n*	%/Mean (SD)/Median (Range)	*n*	%/Mean (SD)/Median (Range)	
Sex, *n* (%)					
Female	22	51.2	26	60.5	0.515
Male	21	48.8	17	39.5	
Age at surgery, years, mean (SD)	36	57.42 (15.78)	34	60.04 (19.20)	0.603
Duration of glaucoma, years, mean (SD)	41	10.03 (10.86)	37	18.18 (14.88)	0.008
Number of previous surgeries, median (range)	42	0.00	40	2.00 (1.00–6.00)	<0.001 ^1^

^1^ Groups compared with chi-square test for sex, with *t*-test for age at surgery and duration of glaucoma or Mann-Whitney *U* test for the number of previous surgeries.

**Table 2:** Visual acuity and interocular pressure mean values, median values, standard deviations, and range before and after surgery.

	Naïve Patients (*n* = 43)		Patients with Previous Surgery (*n* = 43)		MD (95% CI) ^2^	*p* ^3^
	Mean (SD)	Median (Range)	Mean (SD)	Median (Range)		
**Visual Acuity (CDVA)**						
Pre–op	0.37 (0.28)	0.30 (0.00–0.90)	0.38 (0.31)	0.30 (0.01–1.00)	0.01(−0.12–0.14)	0.877
Final	0.44 (0.36)	0.40 (0.01–1.00)	0.45 (0.36)	0.40 (0.00–1.00)	0.01(−0.14–0.18)	0.840
Final vs. Pre-op:						
Change	0.08 (0.19)	0.03 (–0.34–0.50)	0.07 (0.19)	0.05 (−0.30–0.68)	−0.01(−0.09–0.08)	0.902
MD (95% CI) ^1^	0.08 (0.02–0.13)		0.07 (0.01–0.14)			
*p* ^4^	0.011		0.024			
**Intraocular Pressure (IOP)**						
Pre–op	25.00 (7.52)	22.00 (18.00–45.00)	25.35 (7.81)	22.50 (18.00–45.00)	0.35(−3.00–3.70)	0.836
Final	16.83 (5.12)	16.00 (9.00–35.00)	17.54 (5.34)	17.00 (10.00–35.00)	0.71(−1.62–3.04)	0.546
Final vs. Pre-op:						
Change	−6.59 (10.26)	−5.00(−36.00–28.00)	−8.00 (8.86)	−7.00(−34.00–3.00)	−1.41(−5.68–2.85)	0.511
Change (%)	−29.4% (20.1%)	–25.0%(−40.9–−16.7%)	−26.5% (25.4%)	–26.1%(−48.3–−7.8%)	2.9(−7.3–13.2%)	0.567
MD (95% CI) ^1^	−6.59(−10.96–−5.73)		−8.00(−10.87–−5.13)			
*p* ^4^	<0.001		<0.001			

SD: standard deviation; Pre-op: preoperatively; MD: mean difference calculated as final level minus preoperative level ^1^ or as Group 2 minus Group 1 ^2^ with 95% confidence interval; independent *t*-test ^3^ or paired *t*-test ^4^.

**Table 3:** Clinical characteristics of the study group.

Characteristic	Naïve Patients (*n* = 43)		Patients with Previous Glaucoma Surgery (*n* = 43)		*p*
	*n*	%/Median (Range)	*n*	%/Median (Range)	
CDVA change, *n* (%)					
Decline	7/42	16.7	8/39	20.5	0.878
No change	10/42	23.8	8/39	20.5	
Increase	25/42	59.5	23/39	59.0	
IOP change, *n* (%)					
Decline	36/41	87.8	31/39	79.5	0.383 ^1^
No change	2/41	4.9	1/39	2.6	
Increase	3/41	7.3	7/39	17.9	
Decline ≥20%	27/41	65.9	22/39	56.4	0.492
Decline <20%	14/41	34.1	17/39	43.6	
IOP final level <18 mmHg	35/41	85.4	24/39	61.5	0.030
Massage recommendation, *n* (%)	15/43	34.9	9/43	20.9	0.229
Needling, *n* (%)	26/37	70.3	28/38	73.7	0.943
Number of needlings	37	1.00 (0.00;4.00)	38	1.50 (0.00;12.00)	0.117 ^2^
Number of drugs before surgery	43	4.00 (0.00;5.00)	43	4.00 (0.00;5.00)	0.280 ^2^
Time to administration of first drug, days	32	21.00 (0.00;429.00)	33	42.00 (0.00;730.00)	0.058 ^2^
Final number of drugs	41	2.00 (0.00;4.00)	41	2.00 (0.00;4.00)	0.186 ^2^
Next procedures, *n* (%)	15/43	34.9	10/43	23.3	0.342
Complications, *n* (%)	10/43	23.3	13/43	30.2	0.626
Reoperations, *n* (%)	3/18	16.7	8/25	32.0	0.309 ^1^

Groups were compared using the chi-square test, Fisher’s exact test ^1^ for nominal variables, and Mann-Whitney *U* test ^2^ for continuous variables.

**Table 4:** Complications that were observed in the participants.

	Group 1 *n* (%)	Group 2 *n* (%)	*p* *
Intraoperative			
Bleeding	-	1 (2.5)	0.662
Postoperative			
Hyphema			
Blood level in AC	2 (4.7)	2 (5.0)	0.877
Erythrocytes in AC	-	-	-
Wound leakage	1 (2.3)	2 (5.0)	0.383
Fibrosis	9 (21.4)	9 (22.5)	0.881
Anterior chamber cells	3 (7.1)	3 (7.5)	0.841
Hypotony			
Until 7 days	-	2 (5.0)	0.071
Until 30 days	-	1 (2.5)	0.392
Until 180 days	1 (2.3)	1 (2.5)	0.901
Choroid detachment	1 (2.3)	3 (7.5)	0.219
Macular edema	1 (2.3)	-	0.413

AC: anterior chamber; P-ExPress: phaco-Express group; P-Trab: phaco-trabeculectomy, group; * x^2^ test.

## Data Availability

Readers can access the data supporting the conclusions of the study upon an e-mail request from the corresponding author. The names and personal data of the participants could not be released due to ethical considerations.

## Supplementary Materials

The following is available online at https://www.mdpi.com/article/10.3390/jcm10194417/s1, Video S1: Implantation of XEN.
